# Dissonance between predicted and actual retirement statuses to address heterogeneous effects of retirement on mental health; evidence from JSTAR

**DOI:** 10.3389/fpubh.2025.1621198

**Published:** 2025-10-24

**Authors:** Wakako Misawa, Hideki Hashimoto

**Affiliations:** ^1^Graduate School of Medicine, University of Tokyo, Tokyo, Japan; ^2^Department of Health and Social Behavior, School of Public Health, University of Tokyo, Tokyo, Japan

**Keywords:** retirement, mental health, older adults, cognitive dissonance theory, social comparison theory, heterogenous effects, Japan

## Abstract

**Introduction:**

Many studies have explored the relationship between retirement and health outcomes but findings are inconsistent, mainly owing to endogeneity in the relationship between retirement decisions and health, and the effect of heterogeneity across retiree attributes. Recent studies indicate that the mental health effects of retirement vary according to the volitionality of retirement choices taking an exogenous shock as an instrument. In this study, we proposed an alternative strategy to address retirement volitionality and effect heterogeneity using social comparison and cognitive dissonance theories, to treat the dissonance between retirement propensity and actual choice behavior.

**Methods:**

A cross-sectional analysis was conducted using data for 1,544 Japanese men aged 60–75 years derived from the Japanese Study of Aging and Retirement. Drawing on social comparison and cognitive dissonance theories, we hypothesized that an individual’s preferred retirement status could be proxied by the predicted likelihood of retirement status determined in the reference population, and regarded the discrepancy between predicted and actual retirement status as the dissonance status of the retirement decision. The predicted retirement status was inferred from the retirement propensity estimated using a logistic regression model that included variables identified in previous studies as associated with retirement. By comparing predicted and actual retirement status, participants were categorized into four groups as follows: “predicted not-retired and actually not-retired” (PN-AN), “predicted retired and actually retired” (PR-AR), “predicted not-retired but actually retired” (PN-AR), and “predicted retired but actually not-retired” (PR-AN). We investigated between-group differences in the prevalence of depressive symptoms using logistic regression analysis.

**Results:**

Compared with PN and AN individuals, those who were actually retired regardless of their predicted status had higher odds ratios for depressive symptoms (1.91 [95% confidence interval: 1.16–3.12] for PR-AR and 1.84 [1.17–2.91] for PN-AR). The results were robust after adjusting for health conditions and social participation.

**Discussion:**

Our findings indicate that retirement per se was related to depressive symptoms but dissonance between actual and predicted retirement statuses did not modify this association.

## 1 Introduction

In many countries, population aging is regarded as both a social challenge and an opportunity for social and economic sustainability (1, 2). Policy discussions to facilitate the participation of older people in the labor force have focused on financial incentives and employability development from both employer and employee perspectives. Such discussions include consideration of the effects of retirement or continued work on health. This issue has attracted much debate about the attractiveness of longer participation in paid labor and its consequences for social security spending. Although there is extensive evidence on the health effects of retirement or continued work, the findings are inconsistent (3–16).

Recent reviews indicate that there are several sources of inconsistency in research findings on this topic. These include the treatment of endogeneity in the relationship between health and paid labor participation choice, targeted outcomes (e.g., mental, physical, and cognitive health), and effect heterogeneity across individual characteristics and different policy environments (such as pension systems) across countries and time periods (3, 5, 7, 9, 17–30).

Previous studies that have focused on heterogeneity in the retirement process have often examined different effects of the voluntariness of retirement decisions on health (3, 4, 9, 17, 30–32). A meta-analysis by Filomena and Picchio indicated that involuntary retirement has a negative effect on health status (9). However, measures of voluntary/involuntary retirement may be susceptible to the endogeneity problem, as some previous studies have relied on subjective responses which include health reason for retirement reasons (31, 33–38). Other studies have used the timing of policy changes or factory closures to assess voluntariness in the context of an external shock (39, 40). Although this may allow researchers to better manage endogeneity problems, such studies have assessed retirees’ responses to external shocks, rather than the volitional expression of their preference for retirement.

Instead, we propose in this study to newly focus on the dissonance between expected and actual retirement statuses. Drawing on the social comparison and cognitive dissonance theories, we hypothesized that individuals foresee their own “retirement status to be” by referring to external factors and comparing themselves to others who have similar attributes, envisioning the expected treatment afforded to that group as an ideal scenario they aspire to achieve (41, 42). These theories also suggest that individuals should make their decision to retire or continue work so as to match their decision with the expected status given their condition and attributes. Otherwise, they may perceive a dissonance between the expected and the chosen statuses, which leads to psychological distress (43). Similarly, according to social identity theory, individuals tend to define themselves within the context of the groups to which they belong, and are motivated to align themselves with the reference status that their affiliated social group is likely to adopt (44).

We emphasize that this focus on the dissonance between expected and actual retirement statuses is not trivial. Previous studies on voluntary retirement have compared health outcomes between individuals who have voluntarily retired and those who have retired as a response to external shock; they have not included non-retired people. Instead, status dissonance would treat four groups, namely, individuals who have retired with/without dissonance between expected and actual statuses, and those still working with/without status dissonance; these groups cover all the different natural retirement processes. Additionally, as we argue in detail below, the propensity of expected dissonance status can be predicted by regressing on external conditions, which could address the endogeneity problem.

The empirical strategy we used in this study can be alternatively interpreted in the context of causal inference based on counterfactual comparison (45), because the propensity of expected status is taken as a counterfactual status. Thus, the comparison of four groups regarding actual and counterfactual statuses allowed us to examine the causal effects of retirement/continued work participation on health outcomes.

## 2 Materials and methods

### 2.1 Empirical strategies for operationalization of retirement status dissonance

As explained above, we drew on social comparison and cognitive dissonance theories to state that (1) individuals would picture one’s preferred status to be retired or not by referring to the behaviors of others with characteristics similar to their own, and (2) the dissonance between expected retirement status and actual status would lead to psychological distress. To address the first hypothesis, we empirically estimated individuals’ propensity to be retired from paid work using the propensity score method (described below). According to the frameworks of social comparison and cognitive dissonance theories, individuals infer their future status by comparing their external circumstances with those of others and envisioning the treatment accorded to groups with similar attributes as an ideal scenario they themselves ought to attain. In other words, such status can be regarded as a personally desirable condition. Accordingly, we posited that an individual’s preferred retirement status may be proxied by the retirement status predicted on the basis of external factors that affect retirement decisions in the reference population. When the predicted retirement status is not in accordance with the actual retirement status, we regarded this discrepancy as constituting the dissonance status between an individual’s retirement preference and their actual choice. Then, to examine the second hypothesis, we categorized sample individuals into four groups according to their actual retirement status (retired or still working) and the dissonance status based on expected retirement status from the obtained propensity (with or without dissonance).

Several factors influencing the decision-making to retire have been reported (46–52) and previous studies examining the health effects of retirement using the propensity score approach have used these factors to exogenously estimate retirement status (27, 53, 54). We followed this approach. Specifically, we estimated the probability of continued paid labor participation by regressing on known factors influencing the decision, such as age, residential region, educational attainment, employment status at age 54 years, having economically dependent children, retirement status of the spouse, annual income and assets, health status, and social participation.

The choice of retirement or employment should be strongly influenced by pension eligibility. In Japan, there are differences in the pension systems applicable to employees and self-employed individuals, leading to variations in the timing of retirement. Self-employed individuals, who typically start receiving pensions later than the eligibility age and receive lower pension amounts, tend to continue paid labor participation until older ages (55). Additionally, the proportion of residents who are retired in Japan differs between rural areas, urban areas, and large cities owing to differences in industrial and economic structures (51, 52, 56). Therefore, we considered that the cutoff points in the estimated probabilities of paid labor participation should be tailored to differences in age, self-employment/employee status, and local region (e.g., small, medium, and large cities).

We sorted the estimated probability of continued work by age, employment status, and region-specific strata and generated the cutoff point of estimated probability to determine expected status (retired or not) by referring to the actual portion of paid labor participation for each stratum as the reference margin. The rationale was that if the retirement decision followed the estimated propensity, the percentile of the probability corresponding to the actual proportion of paid labor participation in the specific strata should discriminate the decision between retirement and continued work participation. This procedure standardized the propensity of retirement or continued work adjusting for background heterogeneity (57). Finally, estimated retirement status was compared with the actual retirement status, and four combined status types were categorized as follows: “predicted not-retired and actually not-retired” (PN-AN), “predicted retired and actually retired” (PR-AR), “predicted not-retired but actually retired” (PN-AR), and “predicted retired but actually not-retired” (PR-AN).

### 2.2 Data and analysis

We conducted a cross-sectional analysis using data from the baseline survey of the Japanese Study of Aging and Retirement (JSTAR), details of which have been previously published (58). Briefly, the survey probabilistically recruited individuals aged between 55 and 75 years, according to residential records in 10 cities across Japan. A total of 7,047 individuals residing in five areas (Adachi Ward, Kanazawa City, Shirakawa Town, Sendai City, and Takikawa Town) were sampled in 2007, with a response rate of 59.1%. In 2009, 2,401 individuals from two areas (Sendai City and Takikawa Town) were sampled, with a response rate of 65.2%, and in 2011, 3,818 individuals from three cities (Chofu, Tondabayashi, and Hiroshima) were sampled, with a response rate of 57.4%. In total, 7,914 individuals were recruited for the baseline survey of each site.

**Figure 1:**
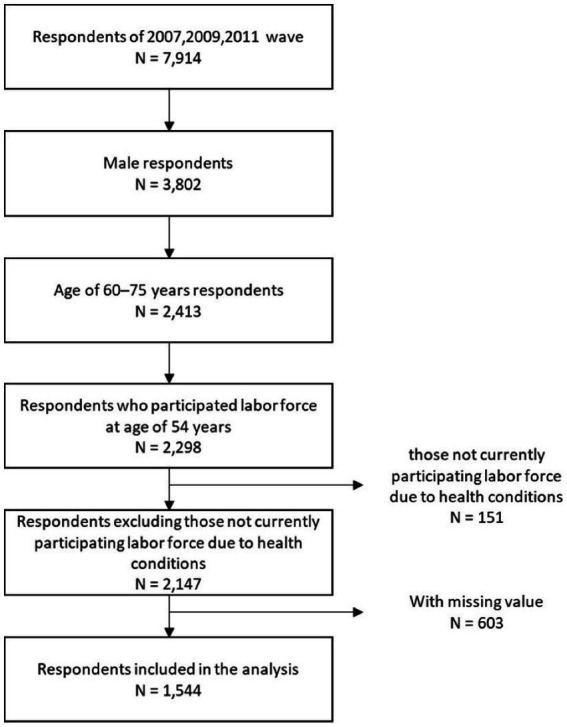
Flow chart describing the sample selection process.

In this study, we analyzed data from 1,544 men aged 60–75 years. The sample selection process for this study is shown in Figure 1. First, among male respondents aged 60–75 years at the time of the survey, the study population was limited to those who reported their labor force participation at the age of 54 years. Next, men who were not participating labor force at the time of the survey because of health reasons were excluded. We also excluded from the analysis individuals with missing data on any of the following items at the time of the survey: employment status, highest level of education, marital status, annual income, number of diagnosed diseases, instrumental activities of daily living (IADL), mobility, and social participation.

#### 2.2.1 Demographic and socioeconomic status

Employees were classified into five age groups: 60–62 years, 63–64 years, 65–67 years, 68–69 years, 70–72 years, and 73–75 years. Self-employed individuals, who comprised a smaller sample, were classified into three age groups: 60–64 years, 65–69 years, and 70–75 years. Urban levels were categorized into major cities (Adachi Ward, Sendai City, Hiroshima City, Kanazawa City), medium-sized cities (Naha City, Chofu City, Tondabayashi City), and rural areas (Takikawa City, Shirakawa City, Tosu City).

Individuals’ highest level of education was categorized as follows: elementary school, middle school, high school, junior college, vocational school, university, and graduate school (master’s and doctoral programs). This information was then dichotomized into “less than university graduate” and “university graduate or higher” for subsequent analyses.

Having economically dependent children may be an incentive to continue work. The number of economically dependent children was counted for each participant, and categorized as “none,” “1,” or “≥2.”

Employment status at the age of 54 years was categorized as “employee” or “self-employed,” and if the individual was an employee at that time, their occupation was categorized as “blue collar,” “white collar,” or “unknown.” Occupation type was categorized based on the major categories of the Japan Standard Occupational Classification; that is, managerial and professional/technical workers, clerical workers, and sales workers were classified as white collar, and all other workers as blue collar (59).

#### 2.2.2 Social participation

Previous research has demonstrated that the extent of social participation in later life is associated with both retirement decision-making and depressive symptoms (60, 61). Social engagement in the previous month was assessed in terms of engagements in community activities (including senior clubs and festivals); community support activities for neighbors; volunteering or charitable activities; hobbies, travel, or entertainment activities; learning or classes; and participation in exercise or sports activities. Individuals who participated in any of these activities were categorized as having social participation (“Yes”).

#### 2.2.3 Health variables

The number of self-reported comorbidities out of listed diagnoses was categorized as “none,” “1,” or “≥2.” Disability in IADL was assessed using a 15-item scale comprising the original 13 items from The Tokyo Metropolitan Institute of Gerontology index of competence, supplemented with two additional items: “ability to make a phone call independently” and “ability to take medication without assistance” (62). Any reported difficulties for one or more of the 15 items was categorized as “limited.” Regarding mobility, participants were categorized as “limited” if they reported difficulties in at least one of 10 mobility-related items such as “Walking 100 meters,” “Sitting in a chair for 2 h,” “Standing up from a chair after sitting for a long time,” and so on. As a simple measure of cognitive function, we used a 10-word recall test; scores were dichotomized into “≤3” and “≥4.”

### 2.3 Outcome

Depressive symptoms were assessed using the 20-item Center for Epidemiological Studies Depression Scale (CES-D), which has established reliability and validity (63, 64). A score of ≥16 was considered to indicate the presence of depressive symptoms. In cases where there were fewer than 10 missing values on the 20 items, the score was calculated by substituting the average values of the responded items.

To investigate the relationship between dissonance in retirement statuses and depressive symptoms, a logistic regression analysis was conducted with the presence or absence of depressive symptoms (1 for presence, 0 for absence) as the dependent variable and dummy variables indicating four groups according to the dissonance status of retirement decision as the independent variables, adjusting for age, residential region, educational attainment, employment status at age 54 years, family member status, economic status, and health status.

These covariates are similar to the variables used to estimate the predicted probability of retirement. Although the predicted retirement status was standardized using these variables, it is reasonable to assume that both the actual retirement status used for the group classification and mental health outcomes are influenced by the same set of variables. Therefore, in the logistic regression analysis for intergroup comparisons, we chose to adjust for these variables as potential confounders. All analyses were conducted using RStudio 2023.06.0 + 421 (The R Foundation for Statistical Computing).

## 3 Results

### 3.1 Characteristics of participants

The descriptive statistics for the 1,544 participants (men aged 60–75 years) are shown in Table 1. The median age of participants was 67 years (interquartile range: 63–70 years). The proportion of paid labor participation at the time of the survey was 59.3%, with 78% of participants being employees and 22% self-employed at the age of 54 years. Additionally, 61.1% of participants were engaged in social participation.

**Table 1:** Descriptive statistics for participant characteristics.

Variable	Category	Value
Age, median (IQR)		67 (63, 70)
Region, *n* (%)	Major city	751 (48.6)
	Medium-sized city	347 (22.5)
	Rural area	446 (28.9)
Highest level of education, *n* (%)	<University graduate	1,159 (75.1)
	≥University graduate	385 (24.9)
Employment status at age 54 years, *n* (%)	Employee	1,205 (78.0)
	Self-employed	339 (22.0)
Spouse’s retirement status, *n* (%)	No spouse	153 (9.9)
	Spouse is retired	864 (56.0)
	Spouse is participating in paid labor	527 (34.1)
Annual income (million yen), median (IQR)		400 (270, 590)
Assets, *n* (%)	<9 million yen	939 (60.8)
	≥9 million yen	538 (34.8)
	Unknown	67 (4.3)
Number of diagnosed diseases, *n* (%)	None	769 (49.8)
	1	474 (30.7)
Presence of IADL limitations, *n* (%)	Not limited	874 (56.6)
	Limited	670 (43.4)
Presence of mobility limitations, *n* (%)	Not limited	1,357 (87.9)
	Limited	187 (12.1)
Number of words recalled, *n* (%)	≥4	1,163 (75.3)
	<3	381 (24.7)
Social participation, *n* (%)	No	601 (38.9)
	Yes	943 (61.1)

IQR, interquartile range; IADL, instrumental activities of daily living.

### 3.2 Estimation of the probability of paid labor participation and grouping of retirement status dissonance

Supplementary Table 1 shows the results of the logistic regression model for paid labor participation. The area under the curve of the model was 0.807. With the obtained model, we estimated for each individual the probability of being in paid labor. The cutoff points by age, region, and work status, which were used to determine expected retirement status, are shown in Supplementary Table 2.

Using the estimated probabilities and cutoffs, each individual was assigned with expected retirement status. When this was compared with their actual status, the data were as follows for the four categories: “predicted retired and actually retired” (PR-AR), 413 individuals (26.7%); “predicted not-retired but actually retired” (PN-AR), 215 individuals (13.9%); “predicted retired but actually not-retired,” 215 individuals (13.9%); “predicted not-retired and actually not-retired” (PN-AN), 701 individuals (45.4%). Table 2 shows the descriptive statistics for the major demographic characteristics and the prevalence of depressive symptoms across the four groups.

**Table 2:** Descriptive statistics for main demographic attributes and prevalence of depressive symptoms across groups based on the dissonance status of retirement decision.

	Groups based on dissonance status of retirement decision				*p*-value
	PR-AR	PN-AR	PR-AN	PN-AN	
Number	413	215	215	701	–
Region, *n* (%)					0.001
Major city	201 (48.7)	90 (41.9)	90 (41.9)	370 (52.8)	
Medium-sized city	92 (22.3)	64 (29.8)	64 (29.8)	127 (18.1)	
Rural area	120 (29.1)	61 (28.4)	61 (28.4)	204 (29.1)	
Age, median (IQR)	70 (67–72)	68 (63–70)	68 (64–71)	64 (62–68)	<0.001
Highest level of education, *n* (%)					0.434
<University graduate university	304 (73.6)	161 (74.9)	155 (72.1)	539 (76.9)	
≥University graduate	109 (26.4)	54 (25.1)	60 (27.9)	162 (23.1)	
Number of economically dependent children, *n* (%)					<0.001
None	333 (80.6)	169 (78.6)	174 (80.9)	551 (78.6)	
1	32 (7.7)	40 (18.6)	18 (8.4)	129 (18.4)	
≥2	48 (11.6)	6 (2.8)	23 (10.7)	21 (3.0)	
Social participation, *n* (%)					<0.001
Yes	209 (50.6)	65 (30.2)	90 (41.9)	237 (33.8)	
No	204 (49.4)	150 (69.8)	125 (58.1)	464 (66.2)	
Employment status at age 54 years, *n* (%)					<0.001
Employee	389 (94.2)	181 (84.2)	181 (84.2)	454 (64.8)	
Self-employed	24 (5.8)	34 (15.8)	34 (15.8)	247 (35.2)	
Type of occupation at age 54 years, *n* (%)					<0.001
Blue collar	115 (27.8)	66 (30.7)	51 (23.7)	183 (26.1)	
White collar	269 (65.1)	95 (44.2)	128 (59.5)	204 (29.1)	
Self-employed	24 (5.8)	34 (15.8)	34 (15.8)	247 (35.2)	
Unknown	5 (1.2)	20 (9.3)	2 (0.9)	67 (9.6)	
Spouse’s retirement status, *n* (%)					<0.001
No spouse	43 (10.4)	20 (9.3)	25 (11.6)	65 (9.3)	
Spouse is retired	326 (78.9)	115 (53.5)	159 (74.0)	264 (37.7)	
Spouse is participating in paid labor	44 (10.7)	80 (37.2)	31 (14.4)	372 (53.1)	
Annual income (million yen), median (IQR)	3.0 (2.3–4.3)	4.3 (2.5–6.6)	3.3 (2.2–4.5)	4.9 (3.3–7)	<0.001
Assets, *n* (%)					0.004
<9 million yen	227 (55.0)	137 (63.7)	117 (54.4)	458 (65.3)	
≥9 million yen	170 (41.2)	70 (32.6)	88 (40.9)	210 (30.0)	
Unknown	16 (3.9)	8 (3.7)	10 (4.7)	33 (4.7)	
Number of comorbidities, *n* (%)					<0.001
None	149 (36.1)	122 (56.7)	84 (39.1)	414 (59.1)	
1	148 (35.8)	57 (26.5)	69 (32.1)	200 (28.5)	
≥2	116 (28.1)	36 (16.7)	62 (28.8)	87 (12.4)	
Presence of IADL limitations, *n* (%)					0.050
Not limited	212 (51.3)	133 (61.9)	121 (56.3)	408 (58.2)	
Limited	201 (48.7)	82 (38.1)	94 (43.7)	293 (41.8)	
Presence of mobility limitations, *n* (%)					<0.001
Not limited	312 (75.5)	204 (94.9)	177 (82.3)	664 (94.7)	
Limited	101 (24.5)	11 (5.1)	38 (17.7)	37 (5.3)	
Number of words recalled, *n* (%)					0.276
≥4	321 (77.7)	156 (72.6)	154 (71.6)	532 (75.9)	
<3	92 (22.3)	59 (27.4)	61 (28.4)	169 (24.1)	
Depressive symptoms, *n* (%)					0.003
No	329 (82.2)	172 (82.3)	189 (90.0)	611 (88.6)	
Yes	71 (17.8)	37 (17.7)	21 (10.0)	79 (11.4)	

IQR, interquartile range; IADL, instrumental activities of daily living; PN-AN, predicted not-retired and actually not-retired; PR-AR, predicted retired and actually retired; PN-AR, predicted not-retired but actually retired; PR-AN, predicted retired but actually not-retired. The *p*-values indicate the results of analyses conducted to examine differences among the four groups based on the dissonance status of retirement decisions, using one-way analysis of variance for continuous variables and the chi-square test for categorical variables.

Regarding age, individuals in the PR-AR group were the oldest, followed by those in the PN-AR and PR-AN groups, with the PN-AN group being the youngest. In terms of employment status, the proportion of employees was relatively high in all groups except for PN-AN, with particularly high proportions of white-collar workers in the PR-AR and PR-AN groups. Concerning the number of comorbidities, limitations in IADL, and mobility restrictions, the PR-AR and PR-AN groups tended to report higher levels of health-related limitations. Finally, the prevalence of depressive symptoms was notably higher in the two groups that were actually retired (i.e., PR-AR and PN-AR).

### 3.3 Relationship between dissonance status of retirement decision and depressive symptoms

Table 3 shows the results of the logistic regression analysis with depressive symptoms as the dependent variable and the dummy variables indicating dissonance status as the independent variables; the reference category was PN-AN (*N* = 701). The odds ratios were 1.91 (95% confidence interval [CI]: 1.16–3.12) for the PR-AR group (*N* = 413), 1.84 (95% CI: 1.17–2.91) for the PN-AR group (*N* = 215), and 0.89 (95% CI: 0.50–1.58) for the PR-AN group (*N* = 215). There was no significant difference in odds ratios for prevalence of depressive symptoms between the two groups who had not actually retired (PN-AN vs. PR-AN group), indicating that retirement dissonance status did not affect depressive symptoms. However, compared with non-retired participants (PN-AN group), those who had retired (PR-AR and PN-AR groups) had significantly higher odds ratios for depressive symptoms.

**Table 3:** Group comparisons of depressive symptoms by dissonance status of retirement decision.

	Estimate	SE	OR	95% CI
Groups based on dissonance status of retirement decision (ref = PN-AN)				
PR-AR	0.645	0.252	1.91	1.16–3.12*
PN-AR	0.612	0.233	1.84	1.17–2.91*
PR-AN	−0.121	0.295	0.89	0.50–1.58
Social participation (ref = Yes)				
No	0.344	0.174	1.41	1.00–1.98*
Employment status at age 54 years (ref = Employee)				
Self-employed	0.216	0.204	1.24	0.83–1.85
Age				
	−0.031	0.020	0.97	0.93–1.01
Highest level of education (ref = <University graduate)				
≥University graduate	−0.159	0.193	0.85	0.58–1.25
Annual income (million yen)				
	−0.009	0.023	0.99	0.95–1.04
Assets (ref = <9 million yen)				
≥9 million yen	−0.268	0.181	0.76	0.54–1.09
Unknown	−0.768	0.483	0.46	0.18–1.20
Number of diagnosed diseases (ref = None)				
1	−0.134	0.187	0.87	0.61–1.26
≥2	0.501	0.197	1.65	1.12–2.43*
Presence of IADL limitations (ref = Not limited)				
Limited	0.582	0.159	1.79	1.31–2.44*
Presence of mobility limitations (ref = Not limited)				
Limited	0.068	0.238	1.07	0.67–1.71

Adjusted for residential region, PN-AN, predicted not-retired and actually not-retired; PR-AR, predicted retired and actually retired; PN-AR, predicted not-retired but actually retired; PR-AN, predicted retired but actually not-retired; IADL, instrumental activities of daily living; SE, standard error; OR, odds ratio; CI, confidence interval. *p*-value < 0.05.

Lack of social participation was related to a higher likelihood of depressive symptoms: odds ratio 1.41 (95% CI: 1.00–1.98). As predicted, having two or more comorbidity conditions (odds ratio 1.65 [95% CI: 1.12–2.43]) and experiencing limitations in IADL (odds ratio 1.79 [95% CI: 1.31–2.44]) were associated with depressive symptoms.

Because of the imbalance in the sample sizes across the four comparison groups, we conducted a sensitivity analysis using bootstrap resampling with replacement for 100 iterations. The resulting odds ratios were very similar to those reported in Table 3, confirming the robustness of our findings.

## 4 Discussion

In this study, we examined the cross-sectional relationship between the dissonance status of retirement decisions and mental health while addressing endogeneity issues and considering heterogeneity based on the social dissonance status of retirement decisions. The results demonstrated that, even after adjusting for confounding factors (including social participation), participants no longer in paid labor showed higher odds ratios for depressive symptoms regardless of the dissonance between actual and predicted work participation status.

### 4.1 Relationship of mental health with dissonance status of retirement decision

The results of this study suggest that retirement itself may be negatively associated with mental health, regardless of whether retirement is anticipated. This finding supports previous studies that have reported a deterioration of mental health in the post-retirement period (13, 65–67) and better mental health in individuals who continue working (15, 68).

Following previous studies suggesting that involuntary retirement negatively affects mental health, we hypothesized that the dissonance in retirement statuses would be negatively associated with mental health. However, the analysis did not support this hypothesis. Instead, our results may indicate that the negative effect of involuntary retirement on mental health may be a consequence of the external shock of having to retire, rather than retirement volitionality per se. Indeed, previous studies have reported that individuals affected by pension reforms experience a deterioration in health as well as financial shocks and abrupt changes in workplace conditions (39, 40). Another possible explanation of our null finding for retirement volitionality relates to social norms regarding retirement in the Japanese cultural context. Specifically, the emphasis in Japan on avoiding “meiwaku” (the concept of inconveniencing or annoying others) often prompts older people to keep themselves socially independent and maintain their contribution to the community, including labor force participation, regardless of their actual preferences and circumstances (69, 70). To address the effects of cultural norms, additional research is needed that incorporates cross-country data and the analytic framework proposed in this study. Our null finding for the volitionality of retirement choice could also be explained by unmeasured confounding, such as individual differences in emotional-cognitive adaptation (71), which require further investigation.

Several previous studies indicate that social participation in later life has beneficial effects on both mental and physical health, and it has been suggested that such participation could mitigate the negative effects of retirement on mental health (13, 72–74). In this study, we adjusted for the presence or absence of social participation, but this did not substantially change the results, suggesting that the continuity of paid labor participation may be independently associated with mental health beyond the effects of other forms of social engagement in the community.

### 4.2 Policy implications

Some countries with population aging challenges, such as Japan, have recently implemented policies aimed to improve national financial sustainability by raising the pension eligibility age and promoting employment among older people, resulting in an increased average effective age of labor market exit (75). Since 2021, employers in Japan have been legally obliged to make good efforts to ensure employment opportunities for people who wish to continue working until the age of 70 years (76). The average effective age of labor market exit for men in Japan as of 2022 was 68.3 years, one of the oldest ages among Organisation for Economic Co-operation and Development countries (75). The findings of the current study may indicate that the current policy direction could help to improve mental health among older men aged 60–75 years in Japan.

### 4.3 Limitations

This study has four limitations. First, as it used a cross-sectional design, causal inferences cannot be drawn. To address the issue of endogeneity between the dissonance status of retirement decision and mental health, we used an analytic strategy similar to the counterfactual comparison of retirement status used by previous studies on the health effects of retirement (27, 53, 54). However, to more fully elucidate the relevant causal relationships, additional longitudinal studies are needed.

The second limitation pertains to generalizability. The study data were derived from Japanese men over a decade ago (2007–2011). In Japan, legislative reforms enacted in 2013 and 2021 have raised the eligibility age for pension benefits while encouraging employers to secure employment opportunities for older adults. Accordingly, the generalizability of our findings may be limited to Japanese men retiring in the post-reform period, who may have a different propensity to retire. Furthermore, as previously mentioned, caution is needed in interpreting the findings because the health effects of retirement can vary across countries (5).

Additionally, gender differences in the retirement process and its effects on health have been documented (7, 17–19, 55). For simplicity, this study focused only on men; retirement for women is a more complex process. Further investigation is necessary to assess these effects in women.

Third, the estimation model for the probability of paid labor participation did not incorporate all variables that are reported to influence retirement decisions, such as workplace condition. However, the logistic regression model used to estimate the probability of paid labor participation had an area under the curve >0.8, suggesting that the predictive accuracy of the model is reasonably strong.

Finally, this study did not account for the number of years since retirement. A previous study using panel data suggested that retirement immediately affected psychological distress, and also affected the pace of post-retirement health changes (12). Although age and health status were included as covariates in the intergroup comparison, it is possible that their influence was not entirely eliminated.

## 5 Conclusion

In this study, we used a propensity score approach to address the issue of endogeneity while accounting for the heterogeneity caused by social dissonance status of retirement to examine the relationship between retirement and mental health. The results indicated a greater likelihood of depressive symptoms in groups that had actually retired, regardless of status dissonance. Retirement itself may have a negative association on mental health, even after adjusting for economic status and social participation.

## Data Availability

The data analyzed in this study is subject to the following licenses/restrictions: application for using a JSTAR dataset to RIETI (The Research Institute of Economy, Trade and Industry). Requests to access these datasets should be directed to https://www.rieti.go.jp/jp/projects/jstar/.
