# Supplementary material for: Dissonance between predicted and actual retirement statuses to address heterogeneous effects of retirement on mental health; evidence from JSTAR
Source: Front Public Health. 2025 Oct 24;13:1621198. doi: 10.3389/fpubh.2025.1621198 (PMC12592161; doi:10.3389/fpubh.2025.1621198)
Supplement: Supplementary file 1 [file Supplementary_file_1.docx]

Supplementary Material

**Supplementary Table 1.** The logistic regression model for the probability of paid labor participation.

|  | **Estimate** | **SE** | **OR** | **95%CI** |  |
| --- | --- | --- | --- | --- | --- |
| **Age** | -0.16 | 0.02 | 0.85 | 0.82–0.88 | * |
| **Highest level of education (ref = <University graduate)** | | | | | |
| ≥University graduate | -0.07 | 0.16 | 0.94 | 0.69–1.27 |  |
| **Number of economically dependent children (ref = None)** | | | | | |
| 1 | 0.28 | 0.18 | 1.33 | 0.93–1.89 |  |
| ≥2 | -0.80 | 0.26 | 0.45 | 0.27–0.75 | * |
| **Employment status at age 54 years (ref = Employee）** | | | | | |
| Self-employed | 1.45 | 0.20 | 4.24 | 2.84–6.34 | * |
| **Type of occupation at age 54 years (ref = Blue collar）** | | | | | |
| White collar | -0.38 | 0.15 | 0.69 | 0.51–0.92 | * |
| Unknown | 0.77 | 0.29 | 2.16 | 1.23–3.80 | * |
| **Spouse's retirement status (ref = No spouse)** | | | | | |
| Spouse is retired | -0.05 | 0.21 | 0.95 | 0.62–1.45 |  |
| Spouse is participating in paid labor | 0.45 | 0.23 | 1.56 | 1.00–2.45 |  |
| **Annual income (million yen)** | 0.15 | 0.03 | 1.16 | 1.10–1.22 | * |
| **Asset (ref = < 9 million yen)** | | | | | |
| ≥9 million yen | -0.30 | 0.14 | 0.74 | 0.56–0.97 | * |
| Unknown | 0.07 | 0.31 | 1.07 | 0.59–1.95 |  |
| **Number of comorbidities (ref = None)** | | | | | |
| 1 | -0.21 | 0.14 | 0.81 | 0.62–1.07 |  |
| ≥2 | -0.36 | 0.16 | 0.70 | 0.51–0.97 | * |
| **Presence of IADL limitations (ref = Not limited )** | | | | | |
| Limited | -0.23 | 0.13 | 0.79 | 0.62–1.02 |  |
| **Presence of mobility limitations (ref = Not limited )** | | | | | |
| Limited | -0.75 | 0.19 | 0.47 | 0.32–0.69 | * |
| **Number of words recalled (ref = ≥4）** | | | | | |
| <3 | 0.03 | 0.14 | 1.03 | 0.78–1.37 |  |
| **Social participation (ref = Yes)** | | | | | |
| No | 0.35 | 0.13 | 1.42 | 1.10–1.84 | * |

Adjusted for residential region

**Supplementary Table 2.** The proportion of paid labor participation and the cutoff points of the estimated probability of paid labor participation

| **Employee at age 54 years** | | | | | |  |  |
| --- | --- | --- | --- | --- | --- | --- | --- |
|  | **Major city** | | **Medium-sized city** | | **Rural area** | | |
| **age-category (years old)** | **The proportion of paid labor participation (%)** | **cutoff** | **The proportion of paid labor participation (%)** | **cutoff** | **The proportion of paid labor participation (%)** | | **cutoff** |
| **60-62** | 83.3 | 0.655 | 81.2 | 0.662 | 73.7 | | 0.734 |
| **63-64** | 69.8 | 0.589 | 56.4 | 0.597 | 72.9 | | 0.612 |
| **65-67** | 55.8 | 0.552 | 38.6 | 0.564 | 47.2 | | 0.598 |
| **68-69** | 46.7 | 0.445 | 45.7 | 0.460 | 52.3 | | 0.463 |
| **70-72** | 22.5 | 0.425 | 42.3 | 0.322 | 29.0 | | 0.375 |
| **73-75** | 13.7 | 0.325 | 22.5 | 0.226 | 40.0 | | 0.286 |

| **Self-employed at age 54 years** | | | | | | |
| --- | --- | --- | --- | --- | --- | --- |
|  | **Major city** | | **Medium-sized city** | | **Rural area** | |
| **age-category (years old)** | **The proportion of paid labor participation (%)** | **cutoff** | **The proportion of paid labor participation (%)** | **cutoff** | **The proportion of paid labor participation (%)** | **cutoff** |
| **60-64** | 96.2 | 0.856 | 90.0 | 0.834 | 87.5 | 0.911 |
| **65-69** | 93.2 | 0.797 | 71.4 | 0.843 | 94.6 | 0.669 |
| **70-75** | 63.0 | 0.653 | 71.4 | 0.589 | 71.4 | 0.606 |
